# Drug Conjugates Such as Antibody Drug Conjugates (ADCs), Immunotoxins and Immunoliposomes Challenge Daily Clinical Practice

**DOI:** 10.3390/ijms131216020

**Published:** 2012-11-28

**Authors:** Wolf-Dieter Janthur, Nathan Cantoni, Christoph Mamot

**Affiliations:** Division of Hematology/Oncology, Cantonal Hospital of Aarau, CH-5001 Aarau, Switzerland; E-Mails: wolf-dieter.janthur@ksa.ch (W.-D.J.); nathan.cantoni@ksa.ch (N.C.)

**Keywords:** targeted therapy, drug conjugates, antibody drug conjugates, immunotoxins, immunoliposomes

## Abstract

Drug conjugates have been studied extensively in preclinical *in vitro* and *in vivo* models but to date only a few compounds have progressed to the clinical setting. This situation is now changing with the publication of studies demonstrating a significant impact on clinical practice and highlighting the potential of this new class of targeted therapies. This review summarizes the pharmacological and molecular background of the main drug conjugation systems, namely antibody drug conjugates (ADCs), immunotoxins and immunoliposomes. All these compounds combine the specific targeting moiety of an antibody or similar construct with the efficacy of a toxic drug. The aim of this strategy is to target tumor cells specifically while sparing normal tissue, thus resulting in high efficacy and low toxicity. Recently, several strategies have been investigated in phase I clinical trials and some have entered phase III clinical development. This review provides a detailed overview of various strategies and critically discusses the most relevant achievements. Examples of the most advanced compounds include T-DM1 and brentuximab vedotin. However, additional promising strategies such as immunotoxins and immunoliposmes are already in clinical development. In summary, targeted drug delivery by drug conjugates is a new emerging class of anti-cancer therapy that may play a major role in the future.

## 1. Background

In the past decade we have seen major advances in the development of suitable, effective and tolerable drug compounds, which aim to deliver drugs more specifically to target tumor cells while sparing healthy tissue. Following the identification of candidate drugs and suitable carrier bonds, the concept of developing drug conjugates to optimize drug effects and patients’ tolerance has progressed from *in vitro* and *in vivo* models to the achievement of promising results in early clinical trials. However, to date, few substances can be considered to be viable options in the daily practice of oncologists or hematologists. Nevertheless, the results of several phase III trials (e.g., ATHERA, MARIANNE, EMILIA and others) have been published (ASCO 2011 and 12) or are underway.

The development of drug conjugates suffered an early setback with the anti-CD33 compound gemtuzumab ozogamicin (Mylotarg^®^). This drug gained accelerated FDA-approval for acute myeloid leukemia (AML) in 2000, but in 2010 (FDA Safety Information, posted June 2010) a confirmatory post-approval trial indicated new safety concerns and failed to demonstrate a benefit, leading to the withdrawal of the product by the manufacturer [1].

Despite this setback, future prospects remain positive, not only for commercially approved drugs, such as the CD30 antibody-cytostatic-complex brentuximab vedotin (SGN 35) [2], but also for others at an advanced stage of development. For example, trastuzumab-emtansine (T-DM1), a conjugate comprising the well-established recombinant humanized antibody trastuzumab (Herceptin^®^) and its cytotoxic partner mertansine [3] has been submitted for regulatory approval. In addition to the antibody drug conjugates (ADCs) other strategies have been devised using different concepts to achieve the same goals. Positive data have been reported for immunotoxins that use targeted carriers to deliver toxins to improve antitumor potency [4]. In particular, there is evidence of activity in hematological tumor types, for example, with the anti-CD22 immunotoxin RFB4(dsFv)-PE38 (BL22) in hairy cell leukemia (HCL) [5]. Another promising strategy is the use of immunoliposomes, which avoid or bypass intracellular drug clearance and enhance intracellular drug concentration by improving internalization into targeted cell structures.

We reviewed the current development status of drug conjugates with regard to their underlying mechanisms, and have summarized the phase III and important phase I/II clinical trials to assess the clinical impact of different strategies.

### 1.1. Reasons to Connect Drugs to Carriers via Linkers

Classical cytotoxic drugs circulate and reach tumor cells at random. Their antitumor effect depends on the higher number of dividing cells in tumors compared with normal tissue. In contrast selective accumulation at the tumor site by targeting specific signs or markers plays a minor role. Since most cytotoxic drugs have a low molecular weight (<1000 g/mol), they rapidly diffuse into tumor cells and healthy tissue. This leads to the known adverse effects, which appear either rapidly or emerge later as delayed toxicity. These unwanted side effects limit the use of potent drugs even if they achieve objective responses and seem beneficial for the patient. In an attempt to improve the efficacy of cytotoxic agents without raising the burden of side effects, researchers have devised strategies to prevent easy diffusion by binding the toxic drugs to macromolecules, such as antibodies, serum proteins, lectins, peptides, growth factors and synthetic polymers.

Although untargeted macromolecules alone are not very specific for tumor cells, they may offer a therapeutic advantage by exploiting the properties of tumor vasculature. The previously described “enhanced permeability and retention (EPR) effect” [6] refers to the increased permeability for macromolecules in tumor tissue vessels, which promotes accumulation [7,8]. An intact endothelial surface prevents the same phenomenon in normal tissues leading to preferential accumulation of cytotoxic agents in tumors [9]. The lack of a proper lymphatic system in malignant tissues also contributes to insufficient drainage and consequent retention of macromolecules in tumors.

There are additional pitfalls for non-specific cytotoxic therapies. For example, they sometimes have to pass boundaries of different pH levels, which leads to either inactivation or activation. Some, such as prodrugs, need to be released or chemically remodeled before becoming active. Often eliminated prematurely before being able to exert their cytotoxic potential, moderately potent chemotherapeutics need to be administered at increased doses and concentrations, leading to the high burden of side effects that the new targeted compounds are designed to avoid.

The design of new drugs or carriers exploits the specific capability of some agents to switch behavior according to their extra- or intracellular location. The process of crossing the cell membrane plays a key role in this respect and should be as specific as possible. By encapsulating or combining with cytotoxic agents, drug carriers can use specific pathways such as receptor mediated-, adsorptive- or fluid-phase endocytosis to deliver the active compound [10].

Turning the spotlight from classic cytotoxic drugs and the need to reduce their inherent disadvantages, the concept of immunotherapy has used receptor-specific antibodies for many years [11]. However immunotherapy seldom achieves complete remissions either used as a single agent or in combination with conventional chemotherapy. While immunotherapy may increase cytotoxic effects by specifically weakening targeted cells [12], this strategy rarely provides adequate single-agent efficacy. The anti-tumor activity of immunotherapy could be improved by coupling the specific immune moiety to a toxic agent to create a new therapeutic entity known as an ADC [13].

Another major problem affecting conventional chemotherapeutics and established immunotherapy is drug resistance. A wide range of resistance mechanisms have been identified including the P-glycoprotein (PGP)-mediated drug efflux and multidrug-resistance protein (MRP), which are both overexpressed drug-export pumps. Other resistance mechanisms include altered folate carriers decreasing the drug uptake, drug inactivation by glutathione-mediated reduction and overexpression of target enzymes [14]. Resistance presents another obstacle that has to be overcome to improve the value of treatment and to increase biological availability to selected cell structures. Several promising liposome-based strategies are being researched with the aim of improving the selective delivery of cytotoxic agents to tumors, including encapsulation of active agents in liposomes to simplify membrane penetration and to reach specific intracellular structures [15,16] and immunoliposomes in which the liposomes carrying the active cytotoxic drug are covalently linked to an antibody fragment with a specific target on tumor cells [17,18].

## 2. Strategies, Carriers, Drugs and Their Linkers

### 2.1. ADCs

Antibodies are well established in modern cancer treatment, often in combination with cytotoxic chemotherapy, and exert their therapeutic effect by multiple mechanisms [19]. However, the activity often seems insufficient and too short lasting [20]. To improve efficacy, researchers devised the promising strategy of linking antibodies to potent cytotoxic agents to combine specificity with the drugs’ activity. This strategy, called conjugation, leads to increased activity of both substances [21], increases drug distribution and reduces harmful exposure of normal tissue. Importantly, conjugation completely alters the pharmacokinetic profile of both components [22]. While additional research is still required to optimize different aspects of the conjugation process, such as drug linkage, Fc-fragments, Fv-targeting, and immunogenicity, it is already evident that some of these immuno-chemo compounds have achieved sustainable and significant clinical success [23].

The affinity of the conjugated antibody for its target should not to be affected or altered compared with its naked or unloaded state. Ideally, the target for the selected antibody should be highly expressed on malignant tissue. To date, successful ADCs comprise two to four potent anticancer small molecule drugs connected to an antibody in random labeling procedures [24]. Meanwhile it was shown, that the chemical and structural properties of the conjugation site of the antibody influence the activity of the conjugate [25]. The narrow therapeutic index achieved with conventional random labeling could be improved even without altering antigen binding by site-specific conjugation [26,27].

The main strategies using antibodies to target cytotoxic agents to malignant cells reported to date, include antibody-protein toxin conjugates or antibody-protein toxin fusion proteins [28], antibody small molecule toxin conjugates [29] and antibody-enzyme conjugates administered with small molecule prodrugs, that require release from the carrier by the conjugated promoter [30].

Connecting the component parts of the drug conjugate is a demanding challenge that influences therapeutic success [31]. The covalent linkers should remain stable in plasma to prevent premature release of the drug, but labile at the target destination, for example, following internalization to liberate the active agent [32]. Linker stability plays a major role in extending the circulating half-life of ADCs to prolong therapeutic effects [33]. Linkers that fulfill these criteria are acid-labile hydrozones, disulfides, thioether and peptides with selective cleavage. Directly linked ADCs and protease-cleavable linkers are preferred to achieve greater stability in the circulation compared with hydrazones and disulfides.

The active drug component needs to possess high anti-tumor potency with a validated mechanism of action, such as DNA damaging and microtubule inhibition. The drugs used in the conjugation process described below proved to be 100–1000-fold more potent in vitro than conventional unconjugated cytotoxics such as taxanes, with IC50 values in the range 0.01–0.1 nM [34]. As mentioned above, many drugs that were too toxic without conjugation showed favorable results as compound partners. Agents fulfilling these criteria and frequently used for conjugation are maytasines [35], calicheamicins and auristatines. Maytasines and auristatines act by binding to a tubulin structure, which consequently leads to G2/M-phase cell-cycle arrest and ends in apoptosis. Monomethylauristatines E and F (vcMMAE/mcMMAF) are synthetic analogs of dolastatin 10, a product of the sea hare *Dolabella ariculara* found in the Indian Ocean [36]. The Ethiopian shrub *Maytenus ovatus* provides the source for the semi-synthetic analogs of maytansines DM1 and 4 [37]. In addition, calicheamicin is a semi-synthetic analog of a fermentation product from *Micromonospora echinospora* ssp. calichensis [38], which is used to create the conjugate gemtuzumab ozogamicin [39,40]. Finally, SN-38, the active drug form of irinotecan (CPT-11), also deserves a mention [41]. The activated form appears to be two to three orders of magnitude more potent than the prodrug [42,43]. SN-38 belongs to the camptothecin group of alkaloids that act as DNA-topoisomerase I-inhibitors.

### 2.2. Immunotoxins

In this review, we define an immunotoxin as a hybrid molecule, constructed by binding a part or all of a toxin to an immunologic ligand, such as a monoclonal antibody or smaller proteins including growth factors and cytokines (IL-2, IL-13, TGFα, GM-CSF), used to destroy tumor cells [44]. It is also necessary to clarify the definition of a “toxin” rather than a cytotoxic drug. Toxin represents any poison produced by an organism, including the bacterial toxins that cause tetanus, diphtheria, *etc.*, and plant and animal toxins such as ricin and snake venom. Immunotoxins derive their potency from the toxin and their specificity from the antibody or alternative transport vehicle [45].

Early examples of immunotoxins were made of plant toxins such as ricin that has the ability to inhibit protein synthesis by interfering with ribosomal RNA [46]. However, limited efficacy and unfavorable vascular damage led to the current standard of genetically altered ricin-A-chains [47]. Other plant-derived toxins used as part of immunotoxin constructs are saporin, gelonin, and poke weed antiviral protein. Recombinant immunotoxins investigated in clinical trials have used two bacterial toxins manufactured as single polypeptide chains: *Pseudomonas aeruginosa* exotoxin A (PE) and diphtheria toxin (DT). These immunotoxins are equipped with three domains for docking onto the target, translocation and catalyzing the ADP-ribosylation that finally leads to the inhibition of protein synthesis in selected cells [48]. The linkers basically do not differ from those utilized in ADCs. The latest studies in the field of immunotoxins focus on overcoming the immunogenicity of extrinsic toxins, which is sometimes limiting in humans. Therefore, humanized endogenous toxic proteins such as the proapoptotic protein RNase are being tested [49].

### 2.3. Immunoliposomes

While using a similar strategy to ADCs and immunotoxins, immunoliposomes offer some potential advantages by equipping an anticancer agent with an immune-based navigation system and modifying drug kinetics to improve tumor cell penetration [50,51] and achieve higher drug levels in target cells. In an immunoliposome, the liposome membrane-coated vesicles that contain the active cytotoxic drug to prevent loss while circulating are modified by attaching a specific antibody or antibody fragment. This constructionn is designed to assure delivery to the target, avoid cellular drug resistance mechanisms and facilitate intracellular penetration [52]. In classic ADCs and immunotoxins the antibody selects the malignant cell lines by targeting specific antigens. However, it is also possible simply to use the Fv chain instead of the whole structure [53].

**Figure 1:**
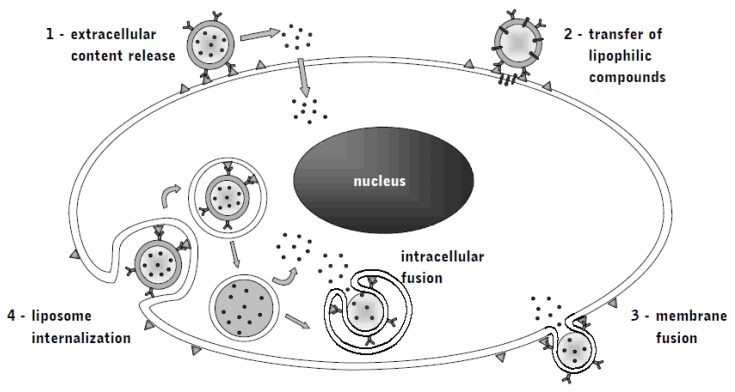
Immunoliposomes: mechanisms of release (**1**), compound transfer (**2**), membrane fusion (**3**) and internalization (**4**)

Liposomes provide stable drug encapsulation and thus long systemic circulation times desirable for use in cancer treatment. For example, pegylation of the single lipid-phosphate bilayer of the liposomal capsule helped to overcome the challenge early bioelimination [54,55]. The previously mentioned EPR-effect in tumors promotes the accumulation of macromolecules in malignant regions, while the non-specific interactions with normal tissue are minimized [56]. Consequently, drug levels at tumor sites and efficacy are increased [57,58]. Other advantages include alternative administration methods and combination with other agents to overcome resistance without altering toxicity (Figure 1).

Delivery of immunoliposomes to tumor cells is facilitated not only by specific recognition of a target, but also by receptor-mediated endocytosis, which can further increase intracellular drug levels and bypasses drug resistance mechanisms such as intracellular drug efflux pumps by altering intracellular drug trafficking. Internalization of the immunoliposome-receptor-complex has been demonstrated for EGFR [59].

The most frequently used drugs in liposomal or immunoliposomal settings are anthracyclines. The first indication for liposomal doxorubicin was Kaposi’s sarcoma in 1997 [60]. Subsequently, they have been approved in many more oncologic settings, mostly gynecologic [61], but also hematologic [62,63], and have also been used in immunoliposomes. Alternative drugs include vincristine [64], paclitaxel [65], bleomycin [66] and different camptothecins [67].

Binding or linkage of liposomes to a specific recombinant antibody or at least fragment of it, is basically carried out via three possible techniques: direct conjugation of the antibody to the liposome (type A), conjugation to liposome and PEG (type B) or attachment to liposome via PEG (type C) [68].

### 2.4. Promising Targets (Antigens)

The molecular target of specifically directed therapies is another key factor for successful achievement of cytotoxic effects. The presence of a candidate molecular target on the surfaces of malignant or other cells or structures serves as a “gateway” to achieve the desired effect of cytoreduction and to create a real synergy [69]. While complete selectivity is practically impossible to achieve, several relevant criteria must be achieved to reach the highest level of selectivity. A high density and concentration of target antigen are very relevant [70]. The target should trigger a distinct immune response to produce a good corresponding antibody. The domain of the antigen location must be accessible and thus on the cell surface. Protein-antigens seem more reactive than others, such as carbohydrates. Accessible tumor-specific antigens or tumor-overexpressed antigens provide the most suitable targets. The higher the overexpression level, the greater the possibility for achieving selectivity [71]. In addition, there is evidence that even the cleavability of the linkers between antibodies and their cytotoxic partner influences the success of target selection and interaction. While cleavable linkers fit a wide range of targets, ADCs with uncleavable linkers have a better profile with respect to the therapeutic window at the target location [72]. The impact of the linker on the efficacy of the whole conjugate was demonstrated by changing only the linkers while keeping the drug composition and target the same [73]. Furthermore, the lipid composition of the immunoliposome has an important effect at the antigen site [74].

In summary, the best activity of targeted therapies is achieved with the highest antigen concentration possible and with antigens as specific as possible. The ideal target is exclusively, or at least mostly, found on tumor cells.

## 3. The Most Promising Conjugates in Clinical Practice

To obtain an overview of substances, compounds, targets and their clinical focus, we have listed them according to their progress and clinical development status (Tables 1–3). In this context, we refer to drugs which are either manufactured as an immunotoxin, ADC or immunoliposome, as defined above. We have also selected the most interesting compounds and their achievements in early and late clinical testing.

### 3.1. Immunoliposomes, New Substances Entering Clinical Stage

The overview of selected trials shows that several agents, especially ADCs, have already entered later clinical development. Neverthelesss, it is also important to highlight even newer, promising substances currently in phase I studies, such as immunoliposomes. Immunoliposomes combine antibody-mediated tumor recognition with liposomal delivery and, when designed for target cell internalization, provide intracellular drug release to increase the specificity and efficacy of the encapsulated drug. Anti-EGFR immunoliposomes are nanoparticles targeting cells expressing the epidermal growth factor receptor (EGFR). It has been shown that doxorubicin-loaded anti-EGFR immunoliposomes (anti-EGFR ILs-Dox) increase the specificity and efficacy of the encapsulated cytotoxic drug. The primary objective of a first-in-human study was to determine the maximum tolerated dose of this new nanocarrier [104]. This compound was constructed by covalently linking pegylated liposomes containing doxorubicin to Fab’ fragments from the monoclonal antibody (Mab) C225 (cetuximab). Escalating doses were administered every 4 weeks for a maximum of six cycles to patients with EGFR-overexpressing advanced solid tumors. Twenty-six patients were treated between January 2007 and May 2010. Interestingly, there were no reports of palmar-plantar erythrodysesthesia, alopecia, cardiotoxicity, or cumulative toxicity. Best response to treatment included one complete response, one partial response, and 10 stable disease lasting 2–12 months (median 5.75 months). In conclusion, anti-EGFR ILs-Dox was well tolerated up to 50 mg doxorubicin/m^2^. Clear evidence of clinical activity was observed warranting further evaluation in phase II trials.

### 3.2. Brentuximab Vedotin, from Phase II into Clinical Practice

The phase II trial table already contains details of exceptional studies that led to early approval of brentuximab vedotin for clinical use. Younes *et al*. confirmed promising results from early stage testing in their pivotal phase II study of brentuximab vedotin for patients with relapsed or refractory Hodgkin’s lymphoma [127]. Brentuximab vedotin (Adcetris^®^) was granted accelerated approval by the FDA in August 2011 on the basis of clinically significant benefit.

The single-arm, open-label, international study analyzed the safety and efficacy of brentuximab vedotin in patients with Hodgkin’s lymphoma who were refractory or relapsed after autologous stem-cell transplantation. The compound’s target, CD30, was histologically proven in the Hodgkin’s lymphoma of the 102 patients receiving brentuximab vedotin at a dose of 1.8 mg/kg every 3 weeks. The study population was rather young, but did include patients up to 77 years of age, reflecting the broad age range within this disease. Most patients were heavily pretreated, having received 3–4 chemotherapy regimens prior to the study. Ninety percent of the patients had received a single autologous stem-cell transplantation and relapsed after a median of 7 months. The ECOG-Score was 0 or 1.

Tumor assessments were independently verified and reported using the Revised Response Criteria for Malignant Lymphoma. CT-scans were performed frequently and supported by PET-scans after 4 and 7 cycles.

Remarkably, 75% of patients responded, almost half of them completely. The remainder achieved stable disease and only three patients progressed. The median duration of response was 6.7 months, with the longest being 14.8 months. The median duration of response in patients meeting the criteria for complete response was approximately 20 months. The overall survival (OS) in this trial reached 22.4 months.

An independent committee also validated safety data. The only grade 4 adverse event reported was neutropenia in six patients. In general, drug-related events were moderate in severity (grades 1 and 2) and therefore manageable. The most common adverse events were nausea/vomiting, diarrhea, fatigue and peripheral sensory neuropathy. Neuropathy affected almost half of the treated population and although reversible in 4/5 of those affected, it led to dose reduction and divergence from the protocol. Neuropathy was attributed to the antimicrotubule component of the compound, as seen with vinca alkaloids. In general, brentuximab vedotin seemed fairly tolerated.

The promising results of this trial challenge previous data with a combination regimen comprising gemcitabine, vinorelbine and pegylated liposomal doxorubicin in the same patient subset [152]. A smaller patient number and fewer complete responses with the combination regimen suggests superior efficacy for brentuximab vedotin, while a comparison of safety profiles explains why brentuximab vedotin provides a new perspective for these heavily treated patients. Hematotoxicity of the combination drug regimen led to grade 3 and 4 adverse events in half of the population.

Brentuximab vedotin does not have a current indication as a first-line therapy and is reserved for a rather rare patient population with few effective therapeutic options. Its safety profile is favorable, highlighting an important issue concerning patients who have already suffered from the effects of demanding cytotoxic therapies in previous lines. The efficacy results also suggest a real improvement in patient outcomes. This pioneering study could open the door to a wider range of indications for brentuximab vedotin and other ADCs.

The confirmatory phase III trial [146] (AETHERA: ADC Empowered Trial for Hodgkin to Evaluate Progression after ASCT) is currently recruiting patients and hopefully will justify the common use of brentuximab vedotin in clinical practice. It is designed as two-arm trial comparing brentuximab vedotin 1.8mg/kg every 3 weeks *vs.* placebo every 3 weeks in patients 30–45 days after autologous stem cell transplantation. Data are to be expected in the following years.

The data obtained with brentuximab vedotin finally provide evidence suggesting the lasting value of therapeutic re-challenge after progression following first-line treatment. At the 2012 ASCO meeting, Bartlett *et al.* presented the first positive results of another phase II study in patients with CD30-positive hematologic malignancies re-treated with brentuximab vedotinin [114]. Treatment was well-tolerated and achieved an objective response rate of 65%.

### 3.3. RFB4-PE38 (BL22), the Most Advanced New Immunotoxin

RFB4 (dsFv)-PE38 (BL22) provides a good example of the immunotoxin subgroup in the ADC class. Kreitman *et al*. have published clinical data on this compound, the most promising from a phase II trial of patients with chemoresistant HCL published in 2009 [129]. The 36 patients enrolled had relapsed or refractory HCL and an indication for re-treatment based on hematologic deficiency. They had all received cladribine and were stratified according to the duration of prior response. All patients received the recombinant anti-CD22 immunotoxin at a dose of 40 μg/kg for three cycles. Treatment was stopped in patients achieving hematologic remission or, in those not in remission, continued at a lower dose of 30 μg/kg for another three cycles.

After one cycle, 25% of the patients achieved a complete remission (CR) and 25% a partial remission (PR). Among the 56% of patients who continued therapy at the lower dose, 47% achieved CR and 25% PR. Serious toxicity was manifest as hemolytic uremic syndrome, which was fully reversible. Neutralizing antibodies were detected in 11% of the treatment population. The overall safety profile was acceptable.

The results were better for patients who had maintained at least one year of response after cladribine than for those who relapsed earlier. The best responses were observed when patients started RFB4-PE38 (BL22) treatment before they developed massive splenomegaly.

The phase II trial provided evidence of activity for the anti-CD22 immunotoxin in patients with HCL and confirmed previous results. There seems to be an even wider range of malignancies that may respond to targeted anti-CD22 therapy compared with anti-CD30 strategies. Inotuzumab ozogamicin is another antibody compound being tested in lymphomas and acute lymphocytic leukemia. Promising phase I/II results have been achieved [123] and a phase III trial is underway [149].

The IL-2 diphtheria toxin fusion protein denileukin diftitox (Ontak^®^) is already well established in routine clinical practice following approval by the FDA in 1999 [153]. Its therapeutic value is restricted to the very limited population of patients with late-stage cutaneous T-cell lymphoma refractory to at least two lines of prior therapy and is dependent on the expression of CD25 (a subunit of the IL-2 receptor) on T-cells [154]. In 2010, a phase III trial finally confirmed and justified the ongoing use of the immunotoxin by providing evidence of a progression-free survival (PFS) benefit and durable objective response rate (ORR) of 44% [151]. Several attempts to expand its indication have been attempted or are on its way, including promising ones, but to date this agent remains useful for only a very limited population.

Immunogenicity is a general challenge or pitfall facing all of the immunotoxins described in this review because they all use foreign proteins. The immune system of patients treated with immunotoxins usually reacts by producing neutralizing antibodies against the toxin, which leads to a lower concentration of the active substance and weakens its potential. The aim of current research is, therefore, to produce recombinant immunotoxins, that induce less immunogenicity [155].

### 3.4. Phase III Trials That will Allow ADCs to Enter Routine Clinical Practice

The EMILIA trial provides a good example of a “fast-track” registrational trial for ADCs. At ASCO 2012 Blackwell K. presented the primary results of this trial evaluating trastuzumab emtansine (T-DM1) *vs.* capecitabine and lapatinib in patients with HER2-positive, locally advanced or metastatic breast cancer previously treated with trastuzumab and a taxane [142]. T-DM1, with its well-known component trastuzumab, targets breast cancer cells expressing the growth factor receptor HER2 and combines its activity with the antitumor effect of emtansine.

Patients were randomized to receive either T-DM1 3.6 mg/kg every 3 weeks or capecitabine 1000 mg/m^2^ days 1–14 every 3 weeks and lapatinib 1250 mg daily. All patients had to be confirmed as having HER2-positive breast cancer by immunohistochemistry and FISH, had metastatic disease and had progressed while being treated with or within 6 months of taxanes and tratsuzumab. Study medication was given in both treatment arms until progression. Primary endpoints were PFS, OS and safety. Efficacy endpoints were monitored by independent review.

Of 991 patients included, 978 were stratified for treatment. Disease history and demographic features appeared well balanced. T-DM1 significantly improved PFS compared with capecitabine/lapatinib (9.6 *vs.* 6.4 months, respectively; hazard ratio (HR) = 0.65, *p* < 0.0001) after a median duration of follow up of 12.5 months. While OS in the capecitabine/lapatinib arm was 23.3 months, the median for T-DM1 was not reached at the time of reporting (HR = 0.621; *p* < 0.0005). T-DM1 was associated with grade 3 thrombocytopenia in 13% of the cases and showed no other unexpected safety problems. The control arm was associated with typical capecitabine side effects including diarrhea, vomiting and palmar-plantar erythrodysesthesia. Overall, grade 3/4 adverse events were observed in 40.8% of patients treated with T-DM1 and in 57% treated with capecitabine/lapatinib.

In conclusion, this analysis demonstrated a significant PFS benefit for T-DM1 treatment compared with the combination of capecitabine/lapatinib, which is already approved for patients with HER2-positive metastatic breast cancer after the failure of trastuzumab. Most importantly for previously treated patients in this palliative setting, serious treatment-related side effects appeared to be less frequent with T-DM1 than with capecitabine/lapatinib. The ADC therefore offers a new mode of action and a valuable therapeutic option. Evidence of significant activity in this phase III trial supported by promising phase I/II data encouraged the manufacturer to apply for regulatory approvals in the U.S. and Europe.

Results of trials of T-DM1 in the first-line setting will follow shortly. MARIANNE, the first phase III trial to study an ADC in combination with an antibody has completed recruitment. In this trial patients are randomized to receive either T-DM1, with or without pertuzumab, or trastuzumab plus docetaxel as first-line treatment of HER2-positive, progressive or recurrent locally advanced or metastatic breast cancer [145]. The primary endpoint is PFS. The objective of investigational regimen is to achieve more complete blockade of the HER2 by combining trastuzumab and pertuzumab, which bind to different epitopes on HER2 and act distinctly. The combination of T-DM1 and pertuzumab may achieve even greater cytotoxic activity than trastuzumab and pertuzumab because of the additional antitumor effects of emtansine. Preclinical models and an earlier phase trial confirmed efficacy and acceptable tolerability. Data analyses are ongoing.

As mentioned above, the clinical use of the gemtuzumab ozogamicin remains controversial. The FDA has withdrawn approval, but nevertheless there are promising data to encourage re-evaluation of its use. The ALFA-0701 trial (first published April 2012) is a randomized, open-label, phase III trial by the French group led by S. Castaigne, which evaluated the effects of gemtuzumab ozogamicin on the survival of adult patients with de-novo AML [148]. A total of 280 patients received either five doses of gemtuzumab ozogamicin on days 1, 4 and 7 during induction and day 1 of each of the two consolidation chemotherapy cycles or regular chemotherapy only. The primary endpoint of event-free survival (EFS) was improved at 2 years with gemtuzumab ozogamicin compared with chemotherapy (40.8 *vs*. 17.1%, respectively; HR = 0.58, *p* = 0.0003). OS, a secondary endpoint, was also improved at 2 years with gemtuzumab ozogamicin (53.2 *vs*. 41.9%; HR = 0.69, *p* = 0.0368). There was no increased risk of death from toxicity with gemtuzumab ozogamicin but persistent thrombocytopenia was more common.

Overall, there was a substantial benefit in terms of EFS and OS for the subset of adult patients with untreated AML and, to date, there have been no reports of major adverse events or safety concerns.

## 4. Conclusions and Future Prospects

Developing ADCs seems to be a logical approach to overcome not only scientific but also physical limitations of systemic cytotoxic therapy for malignancies. After years of advances in the field of drug conjugation it has become obvious that a win-win situation is being established with the potential to change, or at least complement the daily practice of hematologists and oncologists. Irrespective of the strategy used to create the conjugate, whether ADC, immunotoxin or immunoliposome, these new substances not only show antitumor efficacy in clinical trials, but also tend to be less toxic than conventional therapy regimens. The hypothesis of combining the specificity of the immune system with more powerful cytotoxics to enhance efficacy and avoid side effects by targeted delivery is supported by work in preclinical cell models and confirmed by the promising results from well-powered late stage clinical trials.

The data provided by the brentuximab vedotin and the EMILIA trials in particular, and supported by a wealth of data from other studies included in this review, indicate that the transformation into clinical practice is taking place right now. ADCs are already available or will be available soon for use outside clinical trials because they have been approved partly or are close to approval by the regulatory authorities.

However, there are challenges remaining. This new class of antitumor drugs, which lacks long-term results and experience in routine practice, is currently limited to discrete niche indications. However, clear advances have been made and we are confident that targeted drug delivery will play a major role in the future.

## Figures and Tables

**Table 1:** Drug compounds and their targeted antigens and tissues in concluded or on-going phase I trials over the last decade.

Compound	Target	Population	Ref.
AVE9633-amide-MCC-DM4	CD33	Myeloid leukemia	[75]
SAR3419-amide-MCC-DM4	CD19	NHL	[76]
IMGN388-amide-MCC-DM4	Integrin	Solid tumors	[77]
BIIB015-amide-MCC-DM4	Cripto	Solid tumors	[78]
MDX-1203- MC-VC-MGBA (duocarmycin)	CD70	Renal cell carcinoma	[79]
1 C1- MC-MMAF (MEDI-547)	EphA2	Solid tumors	[80]
Ki-4.dgA	CD30	Refractory CD30+ HL and NHL	[81]
Lintuzumab-MC-VC-MMAE	CD30	Hematologic malignancies, HL	[82]
Brentuximab vedotin	CD30	HL, ALCL, ATCL	[83]
Brentuximab vedotin	CD30	HL, ALCL, PTCL	[84]
PSMA ADC	PSMA	Prostate cancer	[85]
MN immunoconjugate	MN	Cancer	[86]
IMGN901	CD56	Multiple myeloma, solid tumors	[87,88]
BT-062	CD138	Multiple myeloma	[89,90]
scFv(FRP5)-ETA	ErbB2/HER-2	Advanced Solid tumor	[91]
BAY 79-4620 MN/CA IX	MN Carbonic anhydrase IX	Solid tumor	[92]
AGS-5ME	AGS-5	Prostate, pancreatic, gastric	[93]
AGS-16M8F	AGS-16	RCC	[94]
SGN-75	CD70	NHL and RCC	[95]
Inotuzumab ozogamicin	CD22	NHL	[96]
IMGN242 (huC242-DM4)	CanAg	Solid tumors	[97]
hLL1-DOX (milatuzumab)	CD74	Multiple myeloma	[98]
SS1P (immunotoxin)	Mesothelin	Mesothelin-positive mesotheliomas, and ovarian and pancreatic cancers	[99]
SS1P (immunotoxin)	Mesothelin	Mesothelin-positive mesotheliomas, and ovarian and pancreatic cancers	[100]
MORAb-009	Mesothelin	Mesothelioma, pancreatic cancer and mesothelin positive lung and ovarian cancer	[101]
CRS-207	Mesothelin	Patients with mesothelin-expressing cancers	[102]
BAY 94-9343	Mesothelin	Patients with advanced solid tumors	[103]
Anti-EGFR ILs-Dox	EGFR	Advanced solid tumors	[104]
MCC-465	epitope recognized by GAH	Metastatic or recurrent stomach cancer	[105]
Moxetumomab pasudotox (CAT-8015 or HA22)	CD22	HCL	[106]
VB4-845	EpCAM	Nonmuscle-invasive bladder cancer in BCG-refractory and BCG-intolerant patients	[107]
NBI-3001	IL-4 receptor	RCC and NSCLC whose tumors showed at least 10% IL-4 receptor expression	[108]
IL13PE38QQR	IL13 receptor	Recurrent malignant gliomas	[109]
SGN-10 (BR96 sFv-PE40)	Lewis(Y)	Solid tumors	[110]
VB4-845	EpCAM	Squamous cell carcinoma of the head and neck	[111]
Trastuzumab-DM1	HER-2	Breast cancer	[112]
Denileukin diftitox	IL-2-rec.	Lymphomas expressing IL-2-receptor	[113]

ALCL = anaplastic large-cell lymphoma, ATCL = angioimmunoblastic T-cell lymphoma, BCG = Bacillus Calmette-Guérin, HL = Hodgkin’s lymphoma, NHL = non-Hodgkin’s lymphoma, NSCLC = non-small cell lung cancer, PTCL = peripheral T cell lymphoma, RCC = renal cell carcinoma.

**Table 2:** Conjugated substances in phase II trials with their antigen and population.

Compound	Target	Population	Ref.
Brentuximab vedotin (SGN-35)	CD30	CD30 positive hematologic malignanancies, retreatment	[114]
Brentuximab vedotin (SGN-35)	CD30	Relapsed or refractory systemic ALCL	[115]
Trastuzumab-DM1	HER2	Breast cancer	[116]
CR011-MC-VC-MMAE	GPNMB	Melanoma	[117–120]
HuC242-amide-MCC-DM4	CanAg	Gastric cancer	[121]
HuN901-amide-MCC-DM1	CD56	Multiple myeloma & SCLC	[122]
Inotuzumab ozogamicin	CD22	ALL	[123]
Brentuximab vedotin (SGN-35)	CD30	HL post ASCT	[124]
Brentuximab vedotin (SGN-35)	CD30	Relapsed or refractory ALCL	[125]
MLN-2704	PSMA	Prostate cancer	[126]
Brentuximab vedotin (SGN-35)	CD30	Relapsed or refractory HL	[127]
Trastuzumab-DM1	HER-2	Breast cancer	[128]
RFB4(dsFv)-PE38 (BL22)	CD22	HCL	[129]
Gemtuzumab ozogamicin (Mylotarg)	CD33	Patients 61 years of age and older with AML	[130]
BMS-182248-1	Lewis-Y	Metastatic breast cancer	[131]
Anti-B4-bR	CD19	Relapsed B-cell NHL	[132]
Anti-B4-bR	CD19	Multiple myeloma	[133]
Trastuzumab-DM1	HER-2	Breast cancer	[134]
Denileukin diftitox	IL-2-rec.	Melanoma, stage IV, unresectable	[135]
Denileukin diftitox	IL-2-rec.	Previously treated advanced NSCLC	[136]
Denileukin diftitox	IL-2-rec.	Previously treated indolent NHL	[137]
Denileukin diftitox	IL-2-rec.	Relapsed/refractory T-cell NHL	[138]
Denileukin diftitox	IL-2-rec.	Previously treated CLL	[139]
Denileukin diftitox	IL-2-rec.	Previously treated B-cell NHL	[140]
Denileukin diftitox	IL-2-rec.	Fludarabine-refractory CLL	[141]

ALCL = anaplastic large-cell lymphoma, ALL = acute lymphocytic leukemia, ASCT = autologous stem cell transplant, AML = acute myeloid leukemia, CLL = chronic lymphocytic leukemia, HL = Hodgkin’s lymphoma, NHL = non-Hodgkin’s lymphoma, NSCLC = non-small cell lung cancer, PTCL = peripheral T cell lymphoma, SCLC = small cell lung cancer

**Table 3:** Active, planned and ongoing phase III trials of the most developed compounds.

Study	Compound	Target	Design	Population	Ref.
EMILIA Completed	Trastuzumab emtansine (T-DM1)	HER-2	*Vs*. capecitabine and lapatinib in HER-2-positive advanced or MBC	Breast cancer	[142–144]
MARIANNE (ongoing, but not recruiting participants)	Trastuzumab emtansine (T-DM1)	HER-2	With or without pertuzumab *vs*. trastuzumab plus taxane in advanced or MBC	Breast cancer	[143,145]
AETHERA (currently recruiting participants)	Brentuximab vedotin (SGN-35)	CD30	*Vs*. placebo in patients with HL progressive after ASCT	HL	[146]
Published	Gemtuzumab ozogamicin	CD33	Observation or gemtuzumab ozo. as postremission treatment	AML at 60 years of age or more	[147]
ALFA-0701 (ongoing, but not recruiting participants)	Gemtuzumab ozogamicin	CD33	Standard treatment with or without gemtuzumab ozo. in de novo AML	AML	[148]
INO-VATE ALL STUDY 1022 (planned)	Inotuzumab ozogamicin	CD22	Inotuzumab ozogamicin *vs*. investigator’s choice in patients with relapsed or refractory ALL	ALL	[149]
Published	anti-B4-bR	CD19	Observation or adjuvant treatment with anti-B4-blocked ricin after BMT	DLBCL in CR after ASCT	[150]
Published	Denileukin diftitox	CD25 subunit of IL-2 receptor	Efficacy and safety of two doses denileukin diftitox in patients who have received three prior therapies	CTLC	[151]

ALL = acute lymphocytic leukemia, AML = acute myeloid leukemia, ASCT = autologous stem cell transplant, BMT = bone marrow transplant, CTCL = cutaneous T-cell lymphoma, DLBCL = diffuse large B-cell lymphoma, HL = Hodgkin’s lymphoma, MBC = metastatic breast cancer, NHL = non-Hodgkin’s lymphoma.
